# Autoimmune Hemolytic Anemia: A Challenging Complication of Allogeneic Hematopoietic Stem Cell Transplant

**DOI:** 10.7759/cureus.73752

**Published:** 2024-11-15

**Authors:** Francisco Abrantes, Joana Dias, Carolina Silva, Isabel Esteves, Maria João Palare, Anabela Ferrão

**Affiliations:** 1 Department of Pediatrics, Hospital de Santa Maria, Unidade Local de Saúde Santa Maria, Lisbon, PRT; 2 Department of Pediatrics, Hospital Beatriz Ângelo, Loures, PRT; 3 Department of Pediatrics, Hospital de Viana do Castelo, Viana do Castelo, PRT

**Keywords:** autoimmune hemolytic anemia (aiha), complication of treatment, hematopoietic stem cell transplantation (hsct), management challenges, rituximab therapy

## Abstract

Autoimmune hemolytic anemia (AIHA) is an uncommon but recognized complication of a hematopoietic stem cell transplant (HSCT). Management challenges include the absence of established guidelines and variable response rates to first-line treatments. We present a case series of three patients, all submitted to HSCT for non-oncologic diseases, who developed AIHA as a complication in the following months. All three were resistant to first-line treatments and required several lines of therapy to achieve a hematological response. Although new treatments have been proposed in refractory cases, multicenter studies and management guidelines are warranted to better characterize this disease and help clinicians.

## Introduction

Autoimmune hemolytic anemia (AIHA) after allogeneic hematopoietic stem cell transplant (HSCT) is an uncommon but potentially life-threatening complication, reported to occur in 2%-6% of stem cell transplanted patients [1, 2]. Autoimmune hemolytic anemia can occur isolated or in combination with other immune-mediated cytopenias, such as immune thrombocytopenia (ITP) [3]. The underlying pathophysiology is still not entirely understood. However, it has been suggested that the impairment of immune tolerance mechanisms may contribute to the development of autoimmune cytopenias [2]. Diagnosis can be challenging due to several concomitant factors, namely drug-related toxicity and graft-versus-host disease (GVHD) [4]. Given the rarity of this complication and the incomplete characterization of its pathophysiology, guidelines on treatment approaches and monitoring are not currently available.

We present a case series of three patients who developed AIHA in the months following HSCT. They were all submitted to allogeneic HSCT in the context of non-oncologic diseases, namely severe combined immunodeficiency (SCID), aplastic anemia, and Griscelli syndrome. After a variable period of time, ranging from three to eight months, all of them developed AIHA. Due to the absence of guidelines and the complexity of the disease pathophysiology, therapy leading to complete hematological responses was challenging, and relapses were frequent. A more detailed characterization of each patient's evolution is described in the following paragraphs.

## Case presentation

Description of patient one

A nine-month-old male patient was diagnosed with SCID following hospitalization due to *Pneumocystis jirovecii *pneumonia. Genetic testing revealed a homozygous mutation of the CD3D gene associated with the T-B+NK+ SCID phenotype. Subsequently, at 13 months old, he underwent an unrelated umbilical cord blood transplant (five of six human leukocyte antigen (HLA)-matched alleles). The conditioning regimen consisted of busulfan, 90 mg/h/L, fludarabine, 160 mg/m^2^, and anti-thymocyte globulin (ATG), 10 mg/kg.

Eight months after the transplant, he was admitted to the hospital due to fever with no apparent focus of infection and new-onset hemolytic anemia (minimum value of hemoglobin 3.3 g/dL). Laboratory findings supporting hemolysis included elevated serum lactate dehydrogenase (LDH) and bilirubin, reticulocytosis, and decreased haptoglobin. A direct antiglobulin test (DAT) was positive, with IgG and C3 antibodies found bound to red blood cells, which combined with elevated hemolysis parameters was consistent with the diagnosis of autoimmune hemolytic anemia. He received two red blood cell-compatible transfusions and was treated with methylprednisolone 30 mg/kg per day for three days and intravenous immunoglobulin (IVIG) 1 g/kg. After the increase in hemoglobin levels, the patient was discharged on prednisolone 1.5 mg/kg/day and maintained regular outpatient follow-up appointments (Table 1).

Approximately one year after the diagnosis of AIHA, and while still on corticoid treatment (prednisolone 0.5 mg/kg/day), he had a symptomatic hemolytic relapse. Hemoglobin levels decreased to 8 g/dL. Therapy was escalated, and he was treated once weekly with rituximab 375 mg/m^2^ for a total of four doses, maintaining treatment with prednisolone (Table 1). There was an excellent response to treatment with a considerable improvement in hemoglobin levels (14.8 g/dL), normal haptoglobin levels (127 mg/dL), and a decrease in LDH (now 350 mg/dL and previously to therapy at 1320 mg/dL) and bilirubin (now 0.3 mg/dL and previously to therapy at 1.94 mg/dL). After continued stabilization of hemoglobin levels, prednisolone was gradually and slowly tapered until complete suspension was achieved, four months after treatment with rituximab. Monitoring after therapy with rituximab revealed maintained normal levels of immunoglobulins, with no need for substitution therapy. No infectious complications occurred.

Four months after corticosteroids were discontinued, the patient presented with fatigue, abdominal pain, and jaundice. Laboratory evaluation showed hemoglobin 6.3 g/dL, absolute reticulocyte count 784x10^9/L, total bilirubin 2.85 mg/dL, direct bilirubin 0.22 mg/dl, and LDH 1178 U/L, confirming another hemolytic crisis. No concomitant cytopenias were present. Positive DAT for IgG and C3 supported the diagnosis of warm AIHA. Second-line treatment with mycophenolate mofetil in association with prednisolone was initiated with a favorable hematologic response. A month later, a slow reduction in corticoid dosage was attempted and tolerated. Shortly after, corticosteroids were discontinued with success. The patient, now five years old, maintains monotherapy with mycophenolate mofetil (600 mg/m^2^/dose twice daily) and has not experienced any further relapses (Table 1). His hemoglobin level at the last visit was 12.3 g/dL, and his last follow-up immune workup revealed T lymphocyte subsets (TCD4+ 1622/uL, TCD8+ 1788/uL), B lymphocyte subsets (651/uL), natural killer cells (328/uL), and normal immunoglobulin levels for age. 

**Table 1:** A summary of the patients' descriptions AIHA: autoimmune hemolytic anemia; ATG: anti-thymocyte globulin; HSCT: hematopoietic stem-cell transplant; HLA: human leukocyte antigen; IVIG: intravenous immunoglobulin; MMF: mycophenolate mofetil; SCID: severe combined immunodeficiency

	Patient 1	Patient 2	Patient 3
Clinical condition leading to HSCT	SCID (T–B+NK+ phenotype)	Aplastic anemia	Griscelli syndrome
Type of HSCT and HLA-matched alleles	Unrelated umbilical cord blood transplant (5/6 HLA-matches)	Allogeneic HSCT from HLA-matched sibling donor (10/10 HLA-matches)	Allogeneic HSCT from an unrelated donor (10/10 HLA-matches)
Conditioning regimen	Busulfan, fludarabine, and ATG	Cyclophosphamide and ATG	Busulfan, fludarabine, and alentuzumab
Minimum hemoglobin levels	3.3 g/dL	3.0 g/dL	5.4 g/dL
Time after HSCT until the development of AIHA	8 months	3 months	6 months
First-line treatment for AIHA	Methylprednisolone (30 mg/kg/day for 3 days) and IVIG (1g/kg)	Methylprednisolone (30 mg/kg/day for 3 days), IVIG (1 g/kg) and plasmapheresis	Prednisolone (6 mg/kg/day)
Other therapies used for refractory AIHA	Rituximab 375 mg/m^2^ (1x a week for 4 weeks), prednisolone, and MMF (variable doses in maintenance therapy)	Rituximab 375 mg/m^2^, (1x a week for 4 weeks), prednisolone, and MMF (variable doses in maintenance therapy)	Rituximab 375 mg/m^2^ (1x a week for 4 weeks) and prednisolone (variable doses in maintenance therapy)
Hemoglobin levels at the last visit	12.3 g/dL	15.0 g/dL	13.0 g/dL
Time since AIHA onset	3 years and 7 months	5 years and 3 months	6 months
Current therapy for AIHA	MMF 300 mg 2x a day	None	None

Description of patient two

A 13-year-old male patient with a medical history of aplastic anemia underwent allogeneic HSCT from an HLA-matched sibling donor (10/10 HLA matches). The conditioning regimen administered consisted of cyclophosphamide and ATG. Graft-versus-host disease prophylaxis was carried out with cyclosporine and methotrexate.

In the weeks following the transplant, the child developed several complications, including necrotizing fasciitis with septic shock. *Pseudomonas aeruginosa* was isolated, and he was treated with broad-spectrum antibiotics and surgical debridement. Three months later, following reconstructive surgery with skin grafting, he presented with fever along with a progressive reduction in hemoglobin (minimum value of hemoglobin 3.0 g/dL) and platelet levels (minimum platelet count of 5×10^9/L). An initial evaluation for hemolysis showed elevated LDH and bilirubin. The DAT was positive. Autoimmune bicytopenia possibly accompanied by microangiopathic hemolytic anemia secondary to cyclosporine was suspected at this point. Cyclosporine was discontinued, and the patient started treatment with corticosteroids (methylprednisolone 30 mg/kg for three days), IVIG (1 g/kg), and plasmapheresis (Table 1). Supportive care with red blood cell concentrates and fresh frozen plasma was provided. The patient failed to respond to the described therapy, and, after a multidisciplinary discussion, he was proposed for treatment with weekly administrations of rituximab 375 mg/m^2^. After the second administration, there was a progressive improvement in hematological parameters, and after one week, hemoglobin rose to 9.9 g/dl and platelet count to 75×10^9/L. Taking into account the favorable hematological response and in order not to exacerbate immunosuppression, it was decided to suspend rituximab after the first two administrations. No substitution therapy was needed, and no infectious complications occurred.

One month later, the patient experienced a relapse. Laboratory evaluation at admission showed hemoglobin of 6.0 g/dL, platelet count of 8X10^9/L, total bilirubin of 3.82 mg/dL, LDH of 682 U/L, and haptoglobin lower than 10 mg/dL. The possibility of transplant-associated thrombotic microangiopathy was investigated, but ADAMTS13 activity was normal. The DAT remained positive, with IgG and C3 antibodies found bound to red blood cells, which was consistent with the previous diagnosis of autoimmune cytopenias. Treatment with corticosteroids and immunoglobulin was started, and he completed four additional doses of rituximab. Despite these treatments, there was an initial decrease in hemoglobin (minimum value of 3.0 g/dL). Supportive care with red blood cell concentrates and fresh frozen plasma was again administered as needed in the acute phase.

Considering the failure to respond to the previously prescribed therapeutics for AIHA and autoimmune thrombocytopenia, he initiated treatment with mycophenolate mofetil (1000 mg 12/12 hours) in association with prednisolone (1 mg/kg) (Table 1). After starting this new line of treatment, there was a satisfactory and consistent response with sustained normal levels of hemoglobin and platelets. The patient, now 18 years old, maintains regular follow-up appointments. He has been gradually reducing dosages over the years, without further relapses. Recently, it was possible to suspend both mycophenolate mofetil and prednisolone, and he has been stable since. On his last visit, he had a hemoglobin level of 15.0 g/dL (Table 1).

Description of patient three

A four-month-old boy with no prior medical history presented at the hospital with three days of fever along with irritability and skin pallor. Laboratory evaluation revealed bicytopenia (anemia and thrombocytopenia), elevated C-reactive protein and liver enzyme levels, as well as slight coagulation abnormalities. Physical examination also highlighted a significant splenomegaly and light silvery-colored hair. Despite early initiation of antibiotic therapy with ceftriaxone, there was a progressive clinical and laboratory deterioration. Hemophagocytic lymphohistiocytosis (HLH) was suspected, and additional bloodwork was ordered. Ferritin levels of 30.000 ng/mL and CD25 levels of 47.000 IU/mL, in addition to the patient’s symptoms, met the clinical and laboratory criteria for HLH, confirming the diagnosis.

After clinical stabilization, the patient was evaluated by a multidisciplinary team. Partial albinism with hypopigmentation of the skin and hair, associated with HLH, raised suspicion for Griscelli syndrome. Therapy for HLH was initiated with methylprednisolone (2 mg/kg/day), cyclosporine (2 mg/kg/day, aiming at serum levels around 200 ng/ml), and alemtuzumab (1 mg/kg/day) with a good clinical response. Genetic testing confirmed the diagnosis of Griscelli syndrome, identifying a heterozygous mutation in the RAB27A gene (c.99A>G and c.467+1G>c). The patient was subsequently proposed for allogeneic HSCT from an unrelated donor (10/10 HLA compatibility), which was performed about a month later. The conditioning regimen consisted of busulfan, 90 mg/h/L, fludarabine, 160 mg/m^2^, and alentuzumab, 1 mg/kg (Table 1).

Approximately six months after HSCT, the patient presented at one of the regular evaluations with increased cutaneous pallor and asthenia. Analytical evaluation revealed a decrease in hemoglobin levels to 7.7 g/dL associated with elevated hemolysis parameters (elevated LDH and bilirubin, and low haptoglobin). The DAT was positive for IgG1 and C3d, confirming the diagnosis of AIHA. Therapy with prednisolone (6 mg/kg/day) was initiated but with only a partial response. After a new multidisciplinary meeting, it was decided to add rituximab to the therapy in four weekly doses of 375 mg/m^2^. The patient had an excellent response to therapy, with an increase in hemoglobin levels to 12.4 g/dL and normalization of hemolysis parameters. It was possible to gradually reduce corticosteroid dosage, which was discontinued two months after starting rituximab therapy.

About three months after discontinuation of corticosteroids, a relapse of AIHA was noted, with a decrease in hemoglobin levels to 5.4 g/dL and a new elevation of hemolysis parameters. The DAT remained positive. Therapy with prednisolone was resumed, but re-initiation of corticosteroid therapy was insufficient to control AIHA, so four more doses of rituximab 375 mg/m^2^ were administered once a week with a favorable clinical and hematological response (increase in hemoglobin to 12.3 g/dL). The patient, currently 22 months old, is still undergoing a slow and gradual corticosteroid taper, maintaining stable hemoglobin levels. On his last visit, he had a hemoglobin level of 13.0 g/dL (Table 1). 

## Discussion

We report three cases of autoimmune manifestations, particularly AIHA, diagnosed in the first year after HSCT. New-onset autoimmune conditions after HSCT have been increasingly recognized as a possible complication of this therapy, and treatment is often challenging, as evidenced in the previously described cases [5]. Isolated or combined immune-mediated cytopenias are the most frequent autoimmune diseases reported in these patients and usually occur in the first months after HSCT [2,5].

The pathophysiologic mechanisms underlying the development of AIHA are still under investigation but may reflect a complex combination of factors such as preformed antibodies and immune dysregulation [2,6,7]. Abnormal thymopoiesis and impaired peripheral immune tolerance due to a reduction of functional regulatory T cells contribute to the escape of auto-reactive lymphocytes, leading to immune phenomena [2,8,9]. Conditioning regimens containing ATG, as seen in cases one and two, and alemtuzumab, as used in case three, are particularly associated with T cell depletion [8,9]. A higher incidence of AIHA has also been described in patients undergoing HSCT for non-malignant diseases, as described in all three cases, which could be partially explained by less exposure to immunosuppressive therapy, and in cord blood transplants, as reported in case one [3]. Other risk factors include unrelated donors, repeated HSCT, chronic GVHD, and peripheral stem cell grafts [1,8,10]. Viral infections have also been associated with immune dysregulation and AIHA after allogeneic HSCT [7,8].

Unspecific symptoms and a broad differential diagnosis contribute to a challenging diagnosis [10,11]. Patients present with symptoms and signs of anemia, including pallor, fatigue, tachycardia, and jaundice. Laboratory findings include increased LDH and bilirubin and low haptoglobin. The DAT report is usually positive, as described in all three cases, but a negative DAT does not rule out AIHA [10]. Other causes of hemolysis should be considered and include AB0 blood group incompatibility, transplant-associated thrombotic microangiopathy, treatment-related toxicity, and GVHD [10,11]. Immune thrombocytopenia is a rare complication of HSCT but affects more frequently pediatric patients than adults [9]. The combination of ITP and AIHA, as described in case two, is designated Evans syndrome and has also been associated with regulatory T-cell dysfunction [9,10,12]. Patients may present with bleeding symptoms [10]. Human platelet antibodies may be identified. However, testing is not mandatory, and the interpretation of the results can be challenging due to the limited sensitivity and specificity of the currently available assays [9,10,13].

Guidelines for the management of immune-mediated cytopenias are not currently available, and treatment decisions are largely supported by case series and expert recommendations [8,10,11]. Several treatments may be needed to achieve a consistent hematological response and to overcome relapses, as highlighted by the three cases reported. Steroids and IVIG are frequently used as first-line therapeutic options, but response rates are lower than achieved in primary AIHA [10,11]. Rituximab, a monoclonal antibody directed against the CD20 antigen expressed on B cells, is frequently prescribed in combination with steroids as an alternative treatment and has a reported response rate of approximately 50 to 80% [9,11,14-16]. Improvement after rituximab was observed in all our patients, followed by a relapse a few months later. In refractory patients or relapse cases, other immunosuppressive therapies such as mycophenolate mofetil, cyclosporine, or cyclophosphamide have been described [9,11]. Mycophenolate mofetil is a prodrug of mycophenolic acid, an inhibitor of inosine-5′-monophosphate dehydrogenase, which reduces lymphocyte proliferation [17]. Variable response rates, according to small case series, are described in the literature [11]. In patients one and two, it has been used with favorable results to prevent relapse. New courses of therapy with rituximab are also a possibility to consider in this case, as used in case three.

Emerging targeted therapies acting on plasma cells, such as bortezomib and daratumumab, and on T cell activation pathways, such as sirolimus and abatacept, may be considered in patients who failed to respond to prior lines of therapy [9,11]. Alemtuzumab, a monoclonal anti-CD52 antibody, and eculizumab, a monoclonal antibody that targets complement protein C5, have also been used in selected cases, but data are still limited, particularly in children [10,11]. In severe refractory cases, splenectomy has been reported [8,11]. Plasmapheresis has been used as a temporizing measure in patients with massive hemolysis, as reported in case two, particularly if caused by cold AIHA given the predominant intravascular distribution of IgM [10,11,14]. Supportive care in severe cases of AIHA includes transfusion of irradiated and leukodepleted red blood cell concentrates. Transfusing these patients is often challenging because auto-antibodies are directed against highly prevalent antigens, hindering the identification of compatible red blood cell units [11]. Platelet transfusions and tranexamic acid may be indicated in patients with Evans syndrome and bleeding manifestations. Thrombopoietin receptor agonists may be added in patients with refractory ITP [10].

Special attention should be paid to early recognition and adequate treatment of opportunistic infections, a frequent cause of death in these patients [11]. Late effects of treatments used for AIHA, such as prolonged corticosteroid therapy, should also be monitored [8,18]. 

## Conclusions

The three reported cases adequately show the complexity and challenges related to the treatment of AIHA after allogeneic HSCT. Relapses are frequent, and several lines of treatment are often needed to achieve consistent hematological responses. Despite promising new therapies, this entity remains a treatment challenge and is associated with important morbidity and mortality. Multicenter studies and management guidelines are warranted to better characterize this disease and help clinicians.
